# Mediating Effect of Upper Limb Use on the Relationship Between Upper Limb Performance and Activities of Daily Living: A Longitudinal Mediation Analysis

**DOI:** 10.7759/cureus.30849

**Published:** 2022-10-29

**Authors:** Yuki Hiraga, Toshiharu Hayashi

**Affiliations:** 1 Department of Health Sciences, International University of Health and Welfare Graduate School, Fukuoka, JPN; 2 Department of Occupational Therapy, Fukuoka International University of Health and Welfare, Fukuoka, JPN; 3 Department of Rehabilitation, Fukuoka Rehabilitation Hospital, Fukuoka, JPN

**Keywords:** mediation analysis, activity of daily living, frequency of use, upper limb performance, stroke

## Abstract

Introduction: Upper limb performance, frequency of upper limb use, and psychological factors are associated with activities of daily living (ADLs) after stroke. We performed a mediation analysis to investigate how the frequency of upper limb use and some psychological factors mediate the relationship between upper limb performance and ADLs.

Methods: Twenty-two patients with stroke were included in this longitudinal study. We utilized the frequency of upper limb use outcome measures (amount of use and quality of motion of the motor activity log), psychological factors outcome measures (General Self-Efficacy Scale), upper limb performance outcome measures (Fugl-Meyer Assessment (FMA)), and ADLs outcome measure (Functional Independence Measure (FIM) motor subscale (M)). Mediation analysis with a bootstrap sampling procedure was used to assess the indirect effects.

Results: Mediation analysis showed that the FMA, as measured by the FIM (M), had significant indirect effects on the amount of use (95% bootstrapped confidence interval (CI): 0.36-2.42) and quality of motion (95% bootstrapped CI: 0.06-1.88). The relationship between upper limb performance and ADLs was mediated by the frequency of upper limb use.

Conclusion: Our findings suggest that improving the frequency of upper limb use may accelerate post-stroke recovery.

## Introduction

Stroke has sequelae symptoms, such as long-term movement disorders and disorders of activities of daily living (ADLs) [1]. Of these, more than 85% of patients with post-stroke hemiplegia have been reported to have residual upper limb dysfunction [2]. Therefore, there is an urgent need for measures to prevent upper limb dysfunction in patients with post-stroke hemiplegia.

Upper limb dysfunction is often observed in patients with post-stroke hemiplegia because only the non-paralyzed upper limb is used in daily life; therefore, it is considered that the upper limbs on the paralyzed side are used less frequently, resulting in learning non-use [3]. Furthermore, it has been reported that patients with acute stroke hemiplegia have already performed ADLs with the upper limbs on the non-paralyzed side within 14 days of onset [4]. Therefore, it can be inferred that it is difficult to use the paralyzed upper limb in daily life after a stroke, and a strategy to increase the frequency of using the paralyzed upper limb in daily life is necessary to improve the limb's function.

It has been reported that among patients with post-stroke hemiplegia, the frequency of using the upper limbs on the paralyzed side in the convalescent and chronic phases is highly correlated with the Fugl-Meyer Assessment (FMA), which indicates the degree of upper limb paralysis [5]. In addition, a study investigating the frequency of upper limb use in patients with post-stroke hemiplegia in the chronic phase revealed that upper limb function affects the frequency of use [6]. From these results, it can be inferred that the frequency of upper limb use and upper limb function affects the ADLs of patients with post-stroke hemiplegia. It has also been clarified that self-efficacy is associated with the ADLs of patients with post-stroke hemiplegia [7], with patients with high self-efficacy having high daily living functions and those with low self-efficacy having low daily living functions. Further, self-efficacy has been found to be associated with disability in patients with distal radius fractures [8]. Therefore, improving the upper limb function, frequency of upper limb use, and self-efficacy is necessary to acquire high daily life function in patients with post-stroke hemiplegia.

The transfer package is a strategy for using the paralyzed upper limb in daily life [9]. It is a method developed to help patients with hemiplegia understand the current problems of the upper limbs and transfer the functions of the upper limbs from the unparalyzed side to the paralyzed side [9]. However, while studies to date have compared pre- and post-intervention effects among intervention and control groups, to the best of our knowledge, the improvement in daily life functions according to the amount of change has not been clarified. Intravenous therapy and bed rest are the main treatments in the acute and convalescent stages after stroke, making it challenging to perform ADLs [4]. Among them, patients with hemiplegia with substantial changes in upper limb function, frequency of upper limb use, and self-efficacy are expected to have a high degree of improvement in ADLs.

The frequency of upper limb use and self-efficacy may affect upper limb performance and ADLs in patients after a stroke. However, it is unknown whether the upper limb performance and ADLs of patients with stroke are mediated by the frequency of using the upper limb and self-efficacy. Therefore, we aimed to evaluate upper limb performance and ADLs in patients with stroke to determine whether the frequency of upper limb use and self-efficacy are mediators. We hope that our findings will provide guidance regarding the planning and implementation of occupational therapy for patients after a stroke.

## Materials and methods

Patients who were diagnosed with stroke within 14 days of onset at the Fukuoka Rehabilitation Hospital, Fukuoka, Japan, between July 2019 and July 2022 were included in this study, which followed a longitudinal study design. All patients involved in this study provided informed consent, and the study was approved by the Ethics Review Board of Fukuoka Rehabilitation Hospital (approval number FRH2019-R-057). Exclusion criteria were: (i) patients with a three-digit Japan Coma Scale score consciousness disorder, (ii) patients with a Mini-Mental State Examination (MMSE) score of less than 24 points, (iii) patients with high brain dysfunction, such as aphasia and unilateral spatial neglect, (iv) medically unstable patients, and (v) patients with bone diseases of the upper limbs. We enrolled 38 patients with stroke with upper limb motor paralysis contralateral to the damaged hemisphere. Using magnetic resonance imaging (MRI) or computed tomography (CT), we screened 34 patients, including 18 with right hemisphere damage and 16 with left hemisphere damage. 

Rehabilitation

Physical therapy, occupational therapy, and speech therapy were implemented for post-stroke rehabilitation from the acute stage. Physiotherapy was performed with the aim of improving the voluntary movement of the paralyzed limb and improving basic movements. Occupational therapy was implemented with the aim of improving applied movement such as in daily life. In speech therapy, we tried to improve speech and swallowing function. For a period of 12 weeks, the patients were rehabilitated for 120-180 minutes per day, seven times every week.

Assessment

ADL

Functional Independence Measure (FIM) scores were collected at admission and discharge for all participants. The FIM consists of 13 motor items and five cognitive items and is evaluated on a seven-grade scale from independence to total assistance. The 13 motor tasks range from 13 to 91 points and rate an individual's ability to perform motor ADLs independently [10]. In this study, the FIM motor score (FIM (M)) was calculated.

Upper Limb Performance

The FMA of the upper limb is a stroke-specific impairment scale that is widely accepted as a measure of physical performance after stroke [11]. In this study, we used the upper extremity sub-score of the FMA. The score ranges from 0 to 66, with higher values indicating lower impairment of the upper limb.

Upper Limb Use

The frequency and quality of the paralyzed upper limb use were assessed using the movement items of the Motor Activity Log (MAL)‐14 [12]. For this subscale, the patient evaluates the frequency of use of the paralyzed limb in everyday life, the amount of use (AOU), quality of movement (QOM), and how skillfully a paralyzed limb can be used in daily life on six levels 0 to 5 [13,14].

General Self-Efficacy

The General Self-Efficacy Scale (GSES) was used to assess the outcome of self-efficacy [15]. The scale comprises 10 items with four answer options, ranging from 1 (not at all true) to 4 (completely true). Higher scores indicate higher levels of self-efficacy. A score of 1.0-2.0 represents a low level, 2.1-3.0 represents a medium level, and 3.1-4.0 represents a high level of self-efficacy.

Statistical Analysis

The Shapiro-Wilk test was used for the distribution of normality. A mediation model was created, and mediation analysis was performed to assess the relationship between upper limb performance and ADLs, frequency of upper limb use, and self-efficacy. First, we calculated the amount of change for each of FIM (M), FMA, AOM, QOM, and GSES. Subsequently, we created a mediation model with X- (independent variable: FMA), M- (mediator variable: AOM, QOM, and GSES), and Y (dependent variable: FIM (M)). As per the model described by Baron and Kenny [16], the related of both independent and mediator variables on the dependent variable were evaluated X and Y. The following condition was met to assess mediation: a bootstrap sampling procedure, as recommended for small sample sizes, was used to determine the significance of indirect effects [17]. In the present study, we specified 1,000 bootstrap iterations, as previously described by Mallinckrodt et al. [17]. In the mediation model make used, the bootstrapped values of the 95% confidence interval (CI) were considered significant mediators [18]. For statistical analyses, HAD version 14.8 (Shimizu, Japan) was used [19]. The statistical significance level was set at 0.05.

## Results

Participant characteristics

During the study period, 12 participants withdrew and there were several missing values in the data from post-stroke weeks 2 and 12. Thus, the number of participants included in the final analysis was 22. The mean age, sex, ischemia, hemorrhage, MMSE, and Brunnstrom recovery stage (upper limb and fingers) of the patients are shown in Table 1.

**Table 1:** Participant characteristics Values are expressed as means ± standard deviation MMSE, Mini-Mental State Examination

Variable	n = 22
Age (years)	60.0 ± 14.0
Gender, male/female, n	11 / 11
Affected side, right/left, n	11 / 11
Hand dominance, right/left, n	22 / 0
Stroke type, hemorrhage/infarction, n	8 / 14
MMSE	28.1 ± 1.7
Bruunstrom Recovery Score	
Stage 1, arm/hand	0 / 1
Stage 2, arm/hand	3 / 1
Stage 3, arm/hand	3 / 4
Stage 4, arm/hand	3 / 2
Stage 5, arm/hand	5 / 5
Stage 6, arm/hand	8 / 9

Outcome measures

We investigated the ADLs, upper limb performance, frequency of upper limb use, and patients' self-efficacy at 2 and 12 weeks post stroke (Table 2). Table 2 also shows the amount of change (Δ) in ADL, Δ (upper limb performance), Δ (frequency of use of upper limb), and Δ (self-efficacy) at 2 and 12 weeks post-stroke.

**Table 2:** Result of outcome measure Values are expressed as means ± standard deviation Δ, change (FMA, AOU, QOM, GSES, FIM (M): 12 weeks after stroke - 2 weeks after stroke; FMA, Fugl-Meyer Assessment; AOU, amount of use; MAL, Motor Activity Log; QOM, quality of motion; GSES, General Self-Efficacy Scale; FIM (M), Functional Independence Measure (motor subscale) score

	2 weeks after stroke	12 weeks after stroke	Δ
FMA	47.2 ± 18.5	54.1 ± 15.3	6.9 ± 6.5
AOU via MAL	2.3 ± 1.9	3.0 ± 1.6	0.7 ± 1.0
QOM via MAL	2.2 ± 2.0	2.8 ± 1.6	0.6 ± 0.8
GSES score	9.5 ± 3.2	10.6 ± 3.2	0.9 ± 1.9
FIM (M)	67.0 ± 20.0	78.1 ± 15.0	11.1 ± 13.0

Findings of mediation analysis

We investigated whether the frequency of use of the upper limb and self-efficacy mediated the relationship between upper limb performance and ADLs. The test model is illustrated in Table 3.

**Table 3:** Mediation analysis: the role of frequency of use and self-efficacy as mediator Δ, change (FMA, AOU, QOM, FIM (M): 12 weeks after stroke - 2 weeks after stroke); SE, standard error; BC, bias-corrected; CI, confidence interval; LL, lower limit; UL, upper limit; FMA, Fugl-Meyer Assessment; AOU, amount of use; QOM, quality of motion; GSES, General Self-Efficacy Scale; FIM (M), Functional Independence Measure (motor subscale) score

Path/effect		β	SE	p-value/95% BCCI
a	ΔFMA→ΔAOU	0.882	0.174	0.001
b	ΔAOU→ΔFIM (M)	0.819	0.281	0.001
c	(direct effect) ΔFMA→ΔFIM (M)	0.198	0.369	0.415
c′	(total effect) ΔFMA→ΔFIM (M)	0.701	0.228	0.001
a × b	(indirect effect) ΔFMA→ΔFIM (M)	0.299	0.513	(LL = 0.369, UL = 2.241)
a	ΔFMA→ΔQOM	0.817	0.172	0.001
b	ΔQOM→ΔFIM (M)	0.707	0.342	0.026
c	(direct effect) ΔFMA→ΔFIM (M)	0.123	0.389	0.631
c′	(total effect) ΔFMA→ΔFIM (M)	0.701	0.228	0.001
a × b	(indirect effect) ΔFMA→ΔFIM (M)	0.378	0.477	(LL = 0.061, UL = 1.882)
a	ΔFMA→ΔGSES	0.287	0.030	0.082
b	ΔGSES→ΔFIM (M)	0.132	0.690	0.116
c	(direct effect) ΔFMEA→ΔFIM (M)	0.663	0.231	0.001
c′	(total effect) ΔFMA→ΔFIM (M)	0.701	0.228	0.001
a × b	(indirect effect) ΔFMA→ΔFIM (M)	0.380	0.490	(LL = -0.002, UL = 0.168)

**Figure 1:**
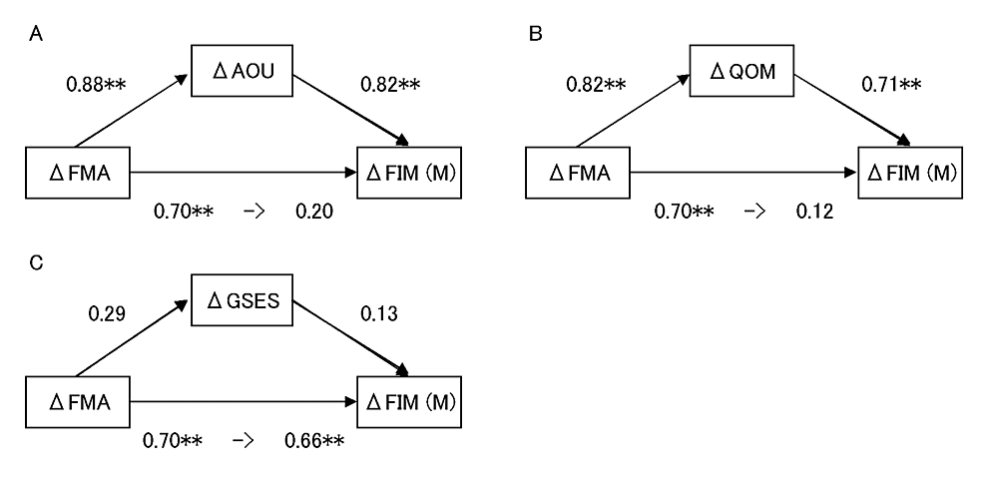
Frequency of use and self-efficacy mediate the relationship between FMA and FIM (M): (A) Amount of use (AOU) is the mediation variable; (B) Quality of motion (QOM) is the mediation variable; (C) General Self-Efficacy Scale (GSES) is the mediation variable Standardized betas are shown; *p < 0.05, **p < 0.01 Δ, change (FMA, AOU, QOM, GSES: 12 weeks after stroke - 2 weeks after stroke); FMA, Fugl-Meyer Assessment; FIM (M), Functional Independence Measure (motor subscale) score

The ΔAOU model results showed a significant relation between ΔFMA and ΔFIM (M), without a mediator. However, there was no significant relation between ΔFMA and ΔFIM (M), although we observed significant relations between ΔFMA and ΔAOU and between ΔAOU and ΔFIM (M), with the introduction of ΔAOU (mediating variable) (Figure 1).

Moreover, we found an indirect effect of ΔFMA and ΔFIM (M) via ΔAOU to have a bootstrap (95%CI, 0.53-2.42) and exhibited a significant effect.

Similarly, we observed a significant relation between ΔFMA and FIM (M), which disappeared with the introduction of ΔQOM (mediating variable) with the ΔQOM model without a mediator. However, we observed significant relations between ΔFMA and ΔQOM and between ΔQOM and FIM (M) (Figure 1). We found the indirect effect of ΔFMA and ΔFIM (M) via QOM to have a bootstrap (95%CI, 0.06-1.88) and exhibited a significant effect.

The ΔGSES model results showed a significant relation between ΔFMA and ΔFIM (M), without a mediator. However, there was no significant association between ΔFMA and ΔGSES and between ΔGSES and ΔFIM (M), although we observed significant positive associations between ΔFMA and ΔFIM (M) (Figure 1), with the introduction of ΔGSES (mediating variable). Moreover, we found the indirect effect of ΔFMA and ΔFIM (M) via ΔGSES to have a bootstrap (95%CI, -0.02-0.16) and exhibited no statistical significance.

## Discussion

We used mediation analysis to investigate the relationship between the FMA of upper limb performance and the FIM (M) of ADLs. The results showed that the relationship between FMA and FIM (M) was indeed fully mediated by AOU and QOM. Cross-sectional studies have shown a relationship between upper limb performance and FIM [20]. Similarly, the present study showed that upper limb performance had a significant effect on FIM (M).

In addition, the results of calculating the amount of change in each measurement index at the time of admission and discharge of patients with post-stroke hemiplegia were 11.1 ± 13.0 for ΔFIM (M), 6.9 ± 6.5 for ΔFMA, 0.7 ± 1.0 for ΔAOU, 0.6 ± 0.8 for ΔQOM, and 0.9 ± 1.9 for ΔGSES. The reported minimal clinically important difference (MCID) values for each of the measures are as follows: FIM (M) = 17 points [21]; FMA = 9-10 points [22]; AOU score = 0.5; and QOM = 0.5-1.1 points [23]. MCID has not been reported for the GSES. The purpose of this study was not to verify the effect of each measurement index by a specific intervention (though the only measurement index that satisfied the MCID criteria was AOU) even if recovery was insufficient. In previous studies, patients were not encouraged to use the upper limbs, because rehabilitation was practiced mainly for restoring function. However, Taub et al. investigated the effect of the transfer package on the frequency of use of paralyzed hands in constraint-induced movement (CIM) therapy using the MAL-AOU, and showed that frequency of use was 2.4 times higher in the group with the transfer package than in the group without [24]. Although all of the participants in this study were right-handed, a previous study reported that upper limb dominance did not interfere with the acquisition of upper limb skills after CIM therapy [25]. Therefore, it is unlikely that upper limb dominance has an effect on MAL outcomes. Given this point, it is necessary to consider the transfer package for CIM therapy.

In this study, we examined whether a change in upper limb performance and a change in the frequency of upper limb use and self-efficacy were related to ADLs after stroke, which has not been investigated in previous studies. Herein, ΔAOU and ΔQOM were identified as factors that mediate ΔFMA and ΔFIM (M) after stroke. It has been reported that upper limb usage improves ADL after stroke [26], and that upper limb function affects the frequency of upper limb use [6]. Furthermore, the relationship between upper limb function and ADL has been clarified in post-stroke hemiplegia [27], with Stevenson et al. reporting that rehabilitation to increase the frequency of upper limb use improves upper limb function and ADL after stroke [28]. Thus, it can be anticipated that patients who have an improvement in upper limb performance will show improved ADL at 12 weeks after stroke, which shall improve further upon frequency of use (AOU and QOM). Additionally, in stroke-affected patients whose upper limb performance has improved, the FIM (M) was affected by mediating the amount of change in the frequency of upper limb use.

Nonetheless, no significant associations were found between ΔFMA and ΔGSES, and ΔGSES and ΔFIM (M) in the present study. Waddell, et al. reported that self-efficacy was not directly related to upper limb function [29]. Therefore, we assumed that a mediation model in which FMA mediates self-efficacy affecting FIM (M) was not established, even in stroke.

This study had several limitations. First of all, the sample size was small; hence, it is possible that the results may vary if the sample size is increased. Second, since the measurement index was based on a questionnaire survey, it was necessary to evaluate it using an activity meter or similar measure. Third, our study could not include neurological factors' relationship between upper limb performance, frequency of use, self-efficacy, and ADLs, because this study did not measure the respective neurotransmitter levels. Further investigations involving larger populations and alternative methods for ADL assessment that would allow for the elucidation of the underlying mechanisms of the relationships found in this study need to be conducted.

## Conclusions

To the best of our knowledge, this is the first study that investigates the mediating effect of frequency of use and self-efficacy on upper limb performance and ADLs following stroke. Our results suggested that AOU and QOM mediate the relationship between upper limb performance and ADLs; therefore, interventions that are focused on improving the frequency of upper limb use may be important in the rehabilitation of patients affected by stroke.
